# Hypokalemia-Induced Arrhythmias and Heart Failure: New Insights and Implications for Therapy

**DOI:** 10.3389/fphys.2018.01500

**Published:** 2018-11-07

**Authors:** Jonas Skogestad, Jan Magnus Aronsen

**Affiliations:** ^1^Division of Cardiovascular and Pulmonary Diseases, Institute of Experimental Medical Research, University of Oslo and Oslo University Hospital, Oslo, Norway; ^2^Department of Pharmacology, Faculty of Medicine, University of Oslo and Oslo University Hospital, Oslo, Norway; ^3^Bjørknes College, Oslo, Norway

**Keywords:** calcium, arrhythmia (heart rhythm disorders), Na^+^ - K^+^-ATPase, hypokalaemia, heart failure

## Abstract

Routine use of diuretics and neurohumoral activation make hypokalemia (serum K^+^ < 3. 5 mM) a prevalent electrolyte disorder among heart failure patients, contributing to the increased risk of ventricular arrhythmias and sudden cardiac death in heart failure. Recent experimental studies have suggested that hypokalemia-induced arrhythmias are initiated by the reduced activity of the Na^+^/K^+^-ATPase (NKA), subsequently leading to Ca^2+^ overload, Ca^2+^/Calmodulin-dependent kinase II (CaMKII) activation, and development of afterdepolarizations. In this article, we review the current mechanistic evidence of hypokalemia-induced triggered arrhythmias and discuss how molecular changes in heart failure might lower the threshold for these arrhythmias. Finally, we discuss how recent insights into hypokalemia-induced arrhythmias could have potential implications for future antiarrhythmic treatment strategies.

## Introduction

Despite continuous improvements in therapies, long-term prognosis in heart failure (HF) remains poor, with overall 5-year mortality reaching 50% (Yancy et al., 2013), and even higher in more advanced stages (NYHA III-IV) (Arnold et al., 2013). Sudden cardiac death (SCD), mostly due to ventricular tachyarrhythmias (VTs), contributes to ~50% of HF deaths (Tomaselli and Zipes, 2004). Hypokalemia is a well-recognized risk factor for VT, and hypokalemia is both common and independently associated with worse clinical outcomes in HF patients (Cleland et al., 1987; Ahmed et al., 2007; Bowling et al., 2010; Kjeldsen, 2010; Aldahl et al., 2017; Nunez et al., 2018), as well as increasing the risk of ventricular arrhythmias and mortality during acute myocardial infarction (Goyal et al., 2012; Colombo et al., 2018; Hoppe et al., 2018).

Here, we review the current evidence for mechanisms of triggered hypokalemia-induced arrhythmias, how cardiac remodeling in HF might lower the threshold for these arrhythmias, and use this to propose future antiarrhythmic drug targets.

### Hypokalemia in HF: etiology and prevalence

Hypokalemia is defined as serum K^+^ levels (serum-[K^+^]) < 3.5 mM (Unwin et al., 2011), but several studies report increased risk of SCD and all-cause mortality in HF patients with serum-[K^+^] <4 mM (Nolan et al., 1998; Macdonald and Struthers, 2004; Bowling et al., 2010; Aldahl et al., 2017). The prevalence of hypokalemia in HF patients varies between 19 and 54% depending on the definition of hypokalemia and patient characteristics (Wester and Dyckner, 1986; Guo et al., 2005; Ahmed et al., 2007; Bowling et al., 2010; Collins et al., 2017). The prevalence was more likely to be higher in patient populations that were studied before the introduction of beta-blockers, ACE-inhibitors, and AT_1_-antagonists as standard HF therapy, as all of these drugs increase serum K^+^ levels and thus counteract hypokalemia. In addition, the prevalence of hypokalemia is generally higher in hospitalized patients compared to nonhospitalized patients (Unwin et al., 2011).

The main causes of hypokalemia in HF are use of diuretics and activation of the renin-angiotensin-aldosterone system that causes loss of K^+^ in the urine (Leier et al., 1994). Increased levels of catecholamines also contribute by shifting K^+^ into the intracellular compartment (Packer, 1990; Osadchii, 2010; Urso et al., 2015), whereas volume overload in more progressive HF could cause a dilution effect (Leier et al., 1994).

It has long been recognized that diuretics, both thiazides and loop-diuretics, increase the risk of hypokalemia and cardiac arrhythmias in patients receiving digitalis (Steiness and Olesen, 1976; Kaplan, 1984). Hypertensive men with baseline ECG abnormalities following an intensive diuretics regime displayed increased mortality compared to the standard regime in the Multiple Risk Factor Intervention Trial (1982). Later trials found no increased mortality with intensive diuretics treatment (1984) or when comparing diuretics to other anti-hypertensive agents (Officers et al., 2002), leading some authors to argue that the anti-hypertensive effect of diuretics compensates for the suggested pro-arrhythmic effect by diuretics alone (Papademetriou, 2006). Nevertheless, one study noted that a minority of patients using thiazides developed marked hypokalemia and cardiac arrhythmias (Siegel et al., 1992), and a case-control study found a dose-response relationship between thiazide dosage and the risk of SCD (Siscovick et al., 1994). Importantly, in patients with left ventricular dysfunction there was 30–40% increased risk of arrhythmic death among patients who used diuretics (Cooper et al., 1999). These results collectively suggest that, even though diuretics are important drugs for blood pressure reduction and prevention of volume overload in HF, being aware of the risk of hypokalemia and cardiac arrhythmias, in particular in the setting of heart disease, is important.

In contrast to thiazides and loop diuretics, mineralocorticoid receptor antagonists limit the renal excretion of K^+^, increase serum-[K^+^], and limit the risk for cardiac arrhythmias induced by hypokalemia (Siscovick et al., 1994; Cooper et al., 1999). ACE inhibitors, aldosterone receptor blockers, and beta blockers could potentially prevent hypokalemia by opposing the neurohumoral activation associated with HF that lowers serum-[K^+^] (Macdonald and Struthers, 2004).

Serum [K^+^] is altered during and after intensive exercise. During exercise, marked hyperkalemia may develop due to the release of K^+^ from skeletal muscles (Sejersted and Sjogaard, 2000). Increased levels of catecholamines counteract and decrease recovery time from exercise-induced hyperkalemia (Williams et al., 1985) by stimulating Na^+^/K^+^-ATPase (NKA) (Despa et al., 2008). Interestingly, serum-[K^+^] undershoots during the recovery phase after physical exercise, leading to postexercise hypokalemia (Medbo and Sejersted, 1990; Lindinger, 1995). The combination of hyperkalemia and subsequent hypokalemia with increased catecholamines during physical exercise could potentially contribute to the increased risk of cardiac arrhythmias and SCD observed during exercise in patients with structural or ischemic heart diseases (Siscovick et al., 1984; Thompson et al., 2007). Intriguingly, the risk of arrhythmias is particularly high in the recovery phase after exercise (Young et al., 1984), which coincides with postexercise hypokalemia (Medbo and Sejersted, 1990; Lindinger, 1995). Low serum-[K^+^] might thus be a cause of arrhythmias in patients even without clinically recognized hypokalemia.

### Triggered ventricular arrhythmias

Ventricular tachyarrhythmias are highly prevalent in HF, with 50–80% of patients having nonsustained VT on ambulatory cardiac monitoring (Singh et al., 1997; Teerlink et al., 2000). Re-entry and triggered arrhythmias are the two main types of tachyarrhythmias (Antzelevitch and Burashnikov, 2011). Fibrosis, scarring, and conduction abnormalities promote mechanical and electrophysiological re-entry, whereas reduced repolarization reserve, Ca^2+^ dysregulation, and altered transmembrane ion currents cause triggered arrhythmias (Tomaselli and Zipes, 2004; Ebinger et al., 2005; Jin et al., 2008).

**Figure 1:**
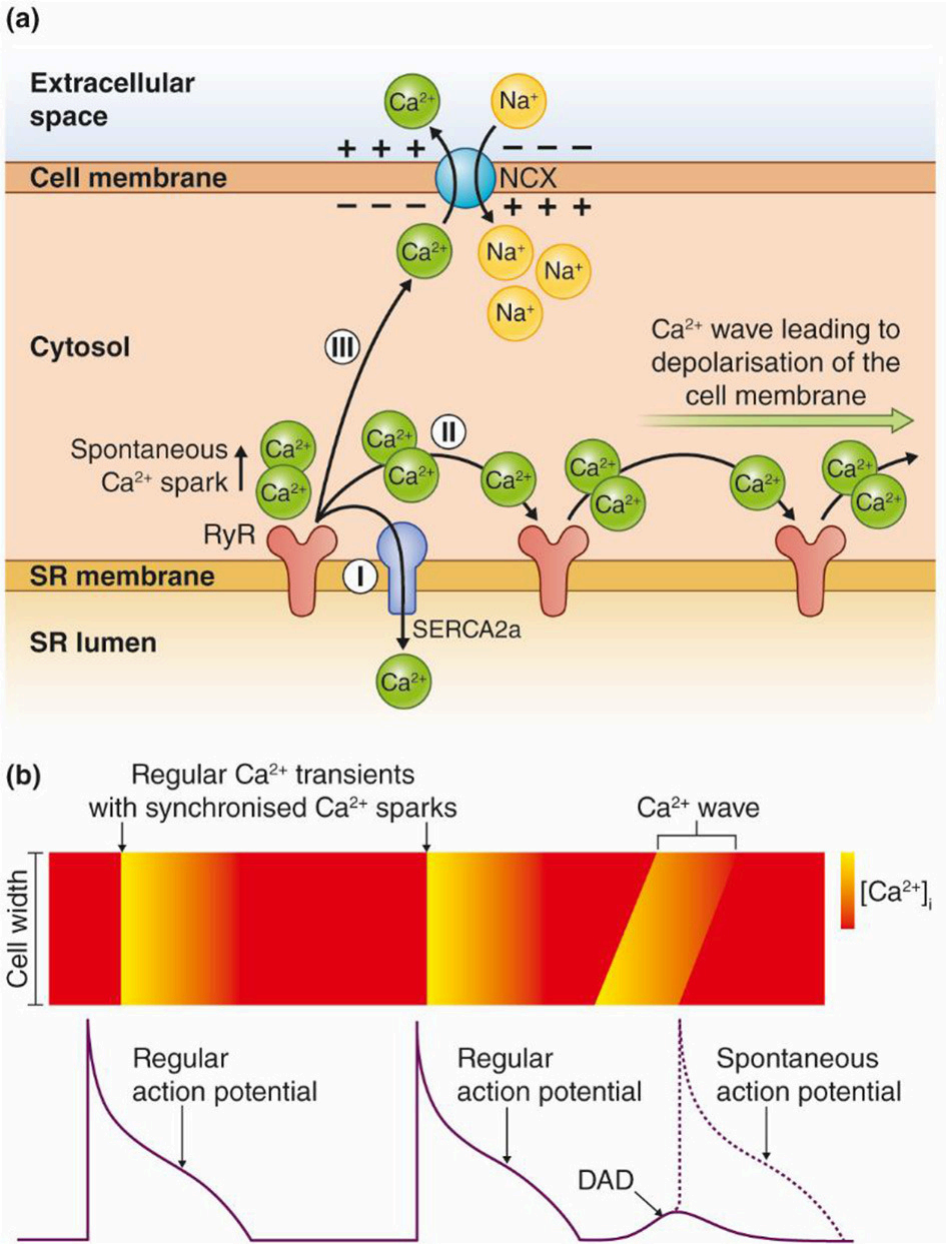
Model for transformation of a Ca^2+^ wave into an afterdepolarization. **(a)** Ca^2+^ ions are released spontaneously from the SR, either due to Ca^2+^ overload or increased RyR conductance (reduced threshold). The Ca^2+^ ions released from RyRs have three possible routes: (I) SERCA2 pumps the Ca^2+^ ions directly back into the SR. The Ca^2+^ wave is interrupted and no afterdepolarizations occur. (II) The Ca^2+^ ions diffuse to the neighboring RyRs, which leads to release of new Ca^2+^ ions. Repetitive events where Ca^2+^ ions are released and activate the next cluster of RyRs along the SR membrane lead to a Ca^2+^ wave. (III) Ca^2+^ can be extruded across the sarcolemma through the Na^+^/Ca^2+^ exchanger (NCX). This causes an inward, depolarizing current due to the inward flux of three positively charged Na^+^ ions per one Ca^2+^ that is extruded over the cell membrane. **(b)** Regular APs trigger synchronous Ca^2+^ release, which leads to cardiomyocyte contraction. Ca^2+^ waves can lead to DADs between two regular APs, and trigger a spontaneous AP as shown in the figure. Ca^2+^ waves during an AP can trigger EADs.

Triggered arrhythmias are reported to initiate most VTs in nonischemic HF and even half of the VTs in ischemic HF (Pogwizd et al., 1992; Pogwizd and Bers, 2004). Triggered arrhythmias are caused by either early or delayed afterdepolarizations (EADs or DADs), abnormal depolarizations of the membrane potential that could give rise to a spontaneous action potential (AP) between two regular APs (Volders et al., 2000). DADs are caused by spontaneous Ca^2+^ release in a feed forward reaction that propagates as Ca^2+^ waves along the sarcoplasmic reticulum (SR) membrane as illustrated in Figure 1. Ca^2+^ waves can occur due to overload of Ca^2+^ in the SR and/or reduced threshold for Ca^2+^ leak through the ryanodine receptors (RyRs) (Venetucci et al., 2008). The spontaneously released Ca^2+^ activates inward currents named I_ti_ (mainly consisting of I_NCX_), leading to a depolarization of the resting membrane potential (Clusin, 2003; Venetucci et al., 2008). The Ca^2+^ wave-induced depolarization triggers an extra AP if the resulting inward current depolarizes the membrane sufficiently to trigger opening of voltage-gated Na^+^ channels.

EADs typically develop in situations with reduced repolarization reserve, either due to increased inward currents, reduced outward currents, or both (Weiss et al., 2010). EADs occur when inward currents, the L-type Ca^2+^ current (I_Ca_) or I_ti_ derived from Ca^2+^ waves during the AP, are larger than the outward currents (mainly K^+^ currents) during late phases of the AP (Zhao et al., 2012).

## Mechanisms for hypokalemia-induced triggered arrhythmias

**Figure 2:**
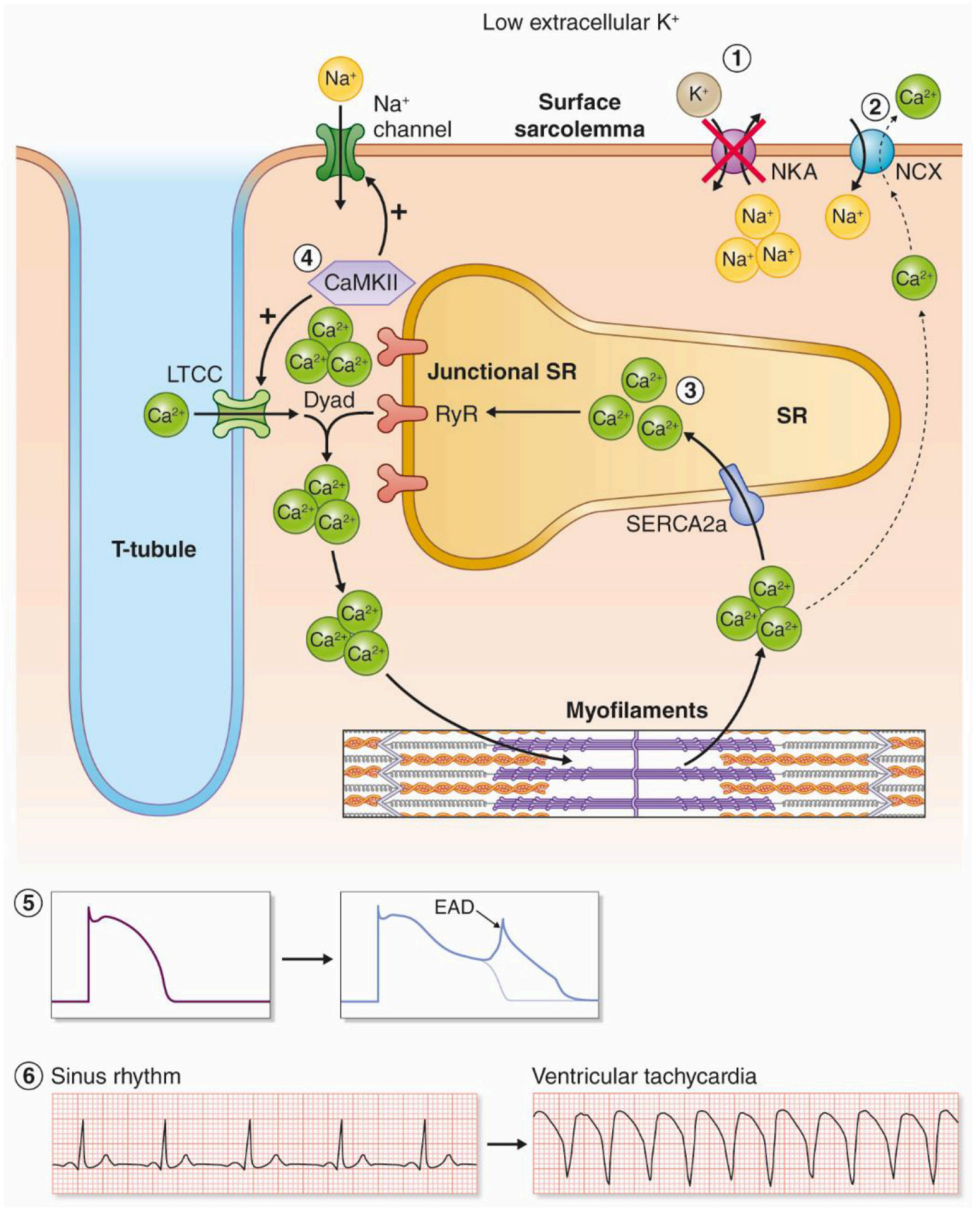
Proposed model for hypokalemia-induced triggered arrhythmias. **(1)** Low ${\text{K}}_{\text{e}}^{+}$ reduces the activity of the NKAα2 isoform. **(2)** Intracellular Na^+^ accumulates and leads to reduced inward NCX current, and by this less extrusion of Ca^2+^. **(3)** Intracellular and SR Ca^2+^ increases as a result. **(4)** Ca^2+^ overload increases the activity of the Ca^2+^/calmodulin-dependent kinase (CaMKII), which leads to a vicious cycle by phosphorylation of voltage-gated Na^+^ channels and L-type Ca^2+^ channels. **(5)** Increased influx of Na^+^ and Ca^2+^ amplifies Ca^2+^ overload and triggers EADs. **(6)** Hypokalemia-induced EADs can trigger ventricular tachyarrhythmias.

Clinically, hypokalemia is associated with triggered arrhythmias such as Torsades De Pointes (TDP), polymorphic VT, ventricular fibrillation (VF), and ventricular ectopy (Nordrehaug et al., 1985). Hypokalemia has been shown to cause regional alterations in conduction velocity (Chah et al., 1982; Smeets et al., 1986; Wolk et al., 1998) and regional action potential duration (APD) heterogeneity (Poelzing and Veeraraghavan, 2007) that establish functional reentry circuits, although a recent study in postinfarction pigs with increased afterload only found slowed conduction velocity with no regional differences in APD (Motloch et al., 2017). Hypokalemia promotes triggered arrhythmias by a reduction in cardiac repolarization reserve and increased intracellular Ca^2+^ in cardiomyocytes (Weiss et al., 2017). Here, we review evidence for mechanisms coupling hypokalemia to induction of triggered arrhythmias, and argue that this is primarily due to inhibition of NKA (in particular the NKAα2 isoform) leading to development of afterdepolarizations as proposed in Figure 2.

### The relative role of NKA vs. K^+^ channels in induction of hypokalemia-induced ventricular arrhythmias

The pro-arrhythmic effects of hypokalemia have been linked to reduced outward K^+^ currents such as I_K1_, I_Kr_, I_Ks_, and I_to_ (Yang et al., 1997; Bouchard et al., 2004; Killeen et al., 2007; Osadchii, 2010) and recently to reduced NKA currents (Aronsen et al., 2015; Pezhouman et al., 2015). Reduced outward K^+^ currents decrease the repolarization reserve, prolong the APD, and increase the risk of afterdepolarizations (Weiss et al., 2017). Low extracellular K^+^ (${\text{K}}_{\text{e}}^{+}$) leads to hyperpolarization of the resting membrane potential, which paradoxically increases excitability of cardiomyocytes. This effect is ascribed to an increased number of available Na^+^ channels and the reduced ability of I_K1_ to generate outward current that protects against membrane depolarization (Bers, 2001).

Pezhouman, Singh, and coworkers observed in an elegant study that reduced NKA activity was necessary and sufficient to develop hypokalemia-induced ventricular arrhythmias. Reduced K^+^ channel conductance in itself caused only a modest increase in APD and no afterdepolarizations, but potentiated the pro-arrhythmic effect of NKA inhibition in this study (Pezhouman et al., 2015). Comparably, we found that NKA inhibition was necessary and sufficient to increase Ca^2+^ levels in a factorial analysis where we compared the relative effect of lowering NKA activity vs. all other ion channels and transporters sensitive to ${\text{K}}_{\text{e}}^{+}$. Several lines of evidence suggest that Ca^2+^ overload caused by NKA inhibition is the main initiating event in hypokalemia-induced ventricular arrhythmias, as discussed in the next sections.

### NCX: the link between NKA inhibition and Ca^2+^ overload in hypokalemia

Na^+^ and Ca^2+^ homeostasis are coupled through the Na^+^/Ca^2+^-exchanger (NCX) that uses the electrochemical gradient of Na^+^ and Ca^2+^ to exchange 3 Na^+^ for 1 Ca^2+^. This allows NKA to indirectly regulate cardiac Ca^2+^ fluxes through the regulation of [Na^+^]_i._ NKA is an ATP- and voltage-dependent ion transporter that exchanges 3 Na^+^ ions from the cytosol with 2 K^+^ ions from the extracellular compartment, leading to a net outward current (Stanley et al., 2015). NKA is the only major Na^+^ efflux mechanism in cardiomyocytes, and regulates intracellular [Na^+^] by balancing Na^+^ efflux against Na^+^ influx (Aronsen et al., 2013; Despa and Bers, 2013).

NKA activity is regulated by extracellular K^+^ levels, intracellular Na^+^ levels, the membrane potential (Glitsch, 2001), posttranslational modifications (Figtree et al., 2009; Poulsen et al., 2010), and the accessory protein phospholemman (PLM), which binds to and inhibits NKA activity (Pavlovic et al., 2007, 2013a). β-adrenergic stimulation leads to phosphorylation of PLM, which relieves PLM inhibition of NKA (Despa et al., 2005; Khafaga et al., 2012). This provides a link between circulating catecholamines and fluxes of Na^+^ and K^+^ over the cell membrane. NKA is composed of αβ dimers, where both isoforms exist in three different isoforms (α1-3 and β1-3) (Lingrel and Kuntzweiler, 1994; Sweadner et al., 1994; McDonough et al., 1996; Bers and Despa, 2009). NKAα1 and NKAα2 contribute to 70–95% and 5–30% of the total NKA activity in cardiomyocytes respectively (Lucchesi and Sweadner, 1991; James et al., 1999; Berry et al., 2007; Despa and Bers, 2007; Swift et al., 2007; Despa et al., 2012). Despite being less abundant than NKAα1, several studies strongly suggest that NKAα2, and not NKAα1, is the main NKA isoform that regulates NCX activity and Ca^2+^ fluxes in cardiomyocytes by limiting the [Na^+^]_i_ sensed by the NCX (Berry et al., 2007; Despa and Bers, 2007; Swift et al., 2007, 2010; Despa et al., 2012). According to this scheme, reduced activity of NKAα2 increases cellular Ca^2+^ levels by limiting forward mode NCX activity (that extrudes Ca^2+^) and by increasing reverse mode NCX activity, which mediates Ca^2+^ influx during a short time during the early phases of the AP (Bers, 2002; Lines et al., 2006).

Despa et al. demonstrated that a similar degree of NKAα1 and NKAα2 inhibition yielded a comparable rise in intracellular Na^+^, but only NKAα2 inhibition increased the Ca^2+^ levels in cardiomyocytes (Despa et al., 2012). This study and others (James et al., 1999; Swift et al., 2007) suggest that the ability of NKAα2 to regulate Ca^2+^ fluxes and cardiac contractility is most likely due to close localization with NCX, but this remains to be shown. NKAα1 is more abundant at the sarcolemma, whereas NKAα2 preferentially localizes to the transverse T-tubules (Berry et al., 2007; Swift et al., 2007; Yuen et al., 2017). The anchoring protein Ankyrin B coordinates a NKA/NCX microdomain, but both NKAα1 and NKAα2 coprecipitate with Ankyrin-B (Mohler et al., 2005). More studies are needed to determine the precise mechanism through which NKAα2 specifically can control Ca^2+^ homeostasis and cardiac contractility.

We studied the effect of low ${\text{K}}_{\text{e}}^{+}$ (2.7 mM) on intracellular Ca^2+^ in rat ventricular myocytes, and found that steady-state Ca^2+^ transients were increased compared to normal K${\text{K}}_{\text{e}}^{+}$ (5.0 mM). The increase in Ca^2+^ was not present in cells pretreated with a low dose of ouabain to selectively inhibit NKAα2 (Aronsen et al., 2015). This result could be explained by the finding that NKAα2 preferentially regulates intracellular Ca^2+^, as discussed above, and the different sensitivity to extracellular K^+^ [K^+^]_e_. Changes in [K^+^]_e_ within the clinical range of hypokalemia are expected to modulate primarily NKAα2 (k_0.5_ = 2.7 mM) and have relatively small effects on NKAα1 (k_0.5_ = 1.5 mM) (Han et al., 2009).

These results suggest that moderate hypokalemia reduces NKAα2 activity and leads to arrhythmogenic Ca^2+^ overload by decreased forward mode and/or reduced reverse mode NCX activity (Aronsen et al., 2015). In addition, chronic hypokalemia has been reported to downregulate the expression of cardiac NKAα2, but not the NKAα1 isoform (Azuma et al., 1991). It cannot be excluded that inhibition of NKAα1 also contributes to the arrhythmogenesis in cells exposed ${\text{K}}_{\text{e}}^{+}$, but given the established role of NKAα2 as a regulator of Ca^2+^ levels in cardiomyocytes, in combination with the present evidence, inhibition of NKAα2 is the most likely mechanism leading to Ca^2+^ overload in hypokalemia.

In addition to NKA inhibition, which reduces inward NCX current, hypokalemia also alters the NCX activity through hyperpolarization of the resting membrane potential. Hyperpolarization increases the inward NCX current and is associated with reduced intracellular Ca^2+^ levels (Bouchard et al., 2004), opposing reduced inward NCX current and the increased intracellular Ca^2+^ following NKA inhibition (Eisner and Lederer, 1979; Aronsen et al., 2013). Both we (Aronsen et al., 2015) and others (Eisner and Lederer, 1979; Bouchard et al., 2004) have reported that myocardial contractility and Ca^2+^ transients are first reduced, and then subsequently increase to above the basal level after switching from normal to low ${\text{K}}_{\text{e}}^{+}$. In the intact organism, changes in ${\text{K}}_{\text{e}}^{+}$ are much slower than in experimental settings, and the Ca^2+^ levels most likely reach steady state without a biphasic response. Still, it is important to appreciate the biphasic cardiomyocyte response to low ${\text{K}}_{\text{e}}^{+}$, as the steady state in myocardium exposed to low ${\text{K}}_{\text{e}}^{+}$ probably reflects the combined effect of membrane hyperpolarization (that reduces Ca^2+^ levels) and NKA inhibition (that increases Ca^2+^ levels).

### Afterdepolarizations due to Ca^2+^ overload in hypokalemia

Two studies have reported Ca^2+^ overload in intact, beating hearts perfused with low K^+^. One study found that the Ca^2+^-induced K^+^ channel was active in hearts exposed to low ${\text{K}}_{\text{e}}^{+}$ (corresponding to clinical hypokalemia) and not in hearts exposed to normal ${\text{K}}_{\text{e}}^{+}$ (Chan et al., 2015). This was further supported in another study that observed that hearts perfused with normal extracellular Ca^2+^ levels, in addition to low ${\text{K}}_{\text{e}}^{+}$, developed VT/VF, but not if the hearts were perfused with low extracellular Ca^2+^. In the latter study, both EADs and DADs appeared in rat hearts exposed to moderate hypokalemia, but only the EADs were followed by episodes of sustained ventricular arrhythmias (Pezhouman et al., 2015). Comparably, only EADs were present in rabbit hearts exposed to moderate hypokalemia (Pezhouman et al., 2015), indicating that that EADs and not DADs are the main trigger of VT/VFs in otherwise normal hearts exposed to hypokalemia.

In addition to the reduction of NKAα2 activity as discussed in the previous sections, other mechanisms contribute to hypokalemia-induced Ca^2+^ overload in the system-based mechanism presented by Pezhouzman, Singh, and coworkers (Pezhouman et al., 2015). First, hypokalemia reduces outward repolarizing currents, both K^+^ currents and the NKA current. This increases the APD, allowing more Ca^2+^ influx through the L-type Ca^2+^ channels during the plateau phase (Weiss et al., 2017). Second, the initial increase in Ca^2+^ caused by NKA inhibition and reduced inward NCX currents, combined with APD prolongation, initiates a positive feedback loop (Pezhouman et al., 2015). According to this model, the initial increase in Ca^2+^ activates CaMKII, which phosphorylates and activates the late Na^+^ current (I_NaL_) and increases I_Ca_. Activation of I_NaL_ and I_Ca_ further amplifies the intracellular overload of Na^+^ and Ca^2+^, eventually activating CaMKII, in a detrimental, downward spiral, ultimately leading to afterdepolarizations and ventricular arrhythmias. Mathematical modeling supported this model, and both CaMKII inhibitors and inhibitor of I_NaL_ prevented development of VT/VF in hearts exposed to hypokalemia. Altogether, the collective data suggest that reduced NKAα2 activity initiates cellular Ca^2+^ overload in hypokalemia, which further leads to ventricular arrhythmias through a feed forward spiral where the activation of CaMKII amplifies intracellular Na^+^ and Ca^2+^ overload, ultimately leading to EADs (Figure 2).

### A possible role for NKA inhibition in other types of ventricular arrhythmias

Some evidence suggests that reduced NKA activity and subsequent development of Ca^2+^ overload and CaMKII activity could be involved in the arrhythmogenesis in other cardiac diseases besides hypokalemia. Transgenic mice with unphosphorylatable PLM have increased incidence of pacing-induced arrhythmias (Pavlovic et al., 2013b), corresponding to the finding that PLM KO have increased amount of triggered arrhythmias after beta-adrenergic stimulation, as PLM phosphorylation protects against Na^+^ and Ca^2+^ overload (Despa et al., 2008). Reduced NKA activity, increased intracellular Na^+^, and reduced inward NCX current are the main mechanisms of digitalis-induced arrhythmias (Wasserstrom and Aistrup, 2005). It has also been reported that digitalis increases CaMKII activity with pro-arrhythmogenic downstream effects, which is a further indication that the pathophysiological mechanisms of digitalis- and hypokalemia-induced arrhythmias are similar (Gonano et al., 2011). The same model could also explain the triggered arrhythmias observed in the Ankyrin B syndrome (LQTS4), a rare genetic syndrome characterized by conductance abnormalities and high risk of ventricular tachyarrhythmias (Cunha and Mohler, 2009). NKA expression is mildly reduced in Ankyrin B^+/−^ mice (Mohler et al., 2003, 2005), and the NKA current is lower than in healthy controls (Camors et al., 2012). Further, forward mode NCX is reduced (Camors et al., 2012), leading to increased CaMKII activity (Popescu et al., 2016) and higher frequency of Ca^2+^ waves (Camors et al., 2012) in Ankyrin B^+/−^ mice, suggesting that the mechanisms discussed for hypokalemia also partly could explain induction of ventricular arrhythmias in the Ankyrin B syndrome.

## Cellular alterations in heart failure and hypokalemia-induced arrhythmias

Molecular remodeling (e.g., transcriptional alterations, posttranscriptional regulation, and posttranslation regulation of proteins) and structural remodeling (cardiac hypertrophy and fibrosis) are hallmarks of HF (Fedak et al., 2005; Kehat and Molkentin, 2010). The remodeling is associated with increased risk of arrhythmias, and might potentiate the pro-arrhythmic effects of hypokalemia. However, there is a paucity of mechanistic data on hypokalemia in HF. Given the high prevalence of hypokalemia in HF patients, more studies are needed to clarify how the remodeling-associated HF influences the risk of hypokalemia-induced arrhythmias. Here, we discuss key alterations in HF that we consider relevant for the expected effect of hypokalemia in failing hearts.

### Factors that promote Na^+^ and Ca^2+^ overload in HF

Intracellular Na^+^ is increased in both human and animal models of hypertrophy and HF (Pieske et al., 2002; Pogwizd et al., 2003), and one study also found increased Na^+^ in the subsarcolemmal space in cells from dogs with cardiac hypertrophy (Verdonck et al., 2003). In theory, the increased Na^+^ levels could be due to increased Na^+^ influx or less Na^+^ extrusion. Although NKA expression and/or activity generally is shown to be reduced in HF, for example by reducing the phosphorylation of PLM (Shamraj et al., 1993; Semb et al., 1998; Bossuyt et al., 2005; Boguslavskyi et al., 2014), one study found no alterations in NKA activity (Despa et al., 2002). Several studies have also reported upregulated I_NaL_ in HF cardiomyocytes (Undrovinas et al., 1999; Moreno and Clancy, 2012), indicating increased Na^+^ influx in HF cardiomyocytes. In a study designed to determine the mechanism for increased Na^+^ concentration in HF, Despa and coworkers found that increased [Na^+^]_i_ in failing rabbit cardiomyocytes (that more closely resembles human cardiomyocytes than rodents) primarily is due to higher TTX-sensitive Na^+^ influx and not due to reduced NKA activity (Despa et al., 2002). Altogether, it seems likely that increased Na^+^ influx and possibly reduced Na^+^ efflux could contribute to the increased intracellular Na^+^ during HF.

Increased intracellular Na^+^ in HF leads to increased intracellular Ca^2+^, by favoring less Ca^2+^ extrusion through forward mode NCX and/or more Ca^2+^ influx through reverse mode NCX. NCX is often upregulated in human (Studer et al., 1994; Reinecke et al., 1996) and experimental (Pogwizd et al., 1999; Sipido et al., 2000) HF, whereas Sarco(Endo)plasmic Reticulum Calcium ATPase 2 (SERCA2) expression and activity are reduced in HF (Lipskaia et al., 2014; Roe et al., 2015). Since intracellular Na^+^ is already higher at baseline and the NCX/SERCA2 balance is shifted in HF, a less pronounced NKA inhibition (e.g., by hypokalemia) could cause sufficient rise in Na^+^ to cause Ca^2+^ overload and spontaneous SR Ca^2+^ release. In addition, one study found pronounced reduction in the NKAα2 isoform expression and function despite only minor changes in the NKAα1 isoform (Swift et al., 2008). Increased expression and activity of CaMKII are also consistently observed in HF (Hoch et al., 1999; Kirchhefer et al., 1999; Zhang et al., 2003), and contribute to increased intracellular Na^+^ and Ca^2+^ by increasing I_Na−L_ (Anderson et al., 2011) and Ca^2+^ leak through RyRs (Ai et al., 2005). On the basis of these observations, we speculate that HF patients are “sensitized” and tolerate less changes in serum-[K^+^] compared to patients without cardiac diseases. This might help explain why HF patients with serum-[K^+^] <4 mM have increased risk of SCD and death (Macdonald and Struthers, 2004; Bowling et al., 2010), compared to the conventional cut-off at 3.5 mM (Unwin et al., 2011).

### Factors that promote afterdepolarizations in HF

Spontaneous Ca^2+^ waves induce DADs by activating an inward current mainly consisting of I_NCX_ (Clusin, 2003). Since NCX typically is upregulated in HF, a given amount of spontaneously released Ca^2+^ generates more depolarizing inward current, reducing the threshold for DADs (Pogwizd and Bers, 2004). I_K1_ acts as a “safety valve” and counterbalances depolarizing inward currents caused by spontaneous Ca^2+^ release during the resting phase of the AP. I_K1_ is downregulated in HF (Fauconnier et al., 2005; Bers, 2006), thus increasing the likelihood that a spontaneous AP occurs through generation of EADs and/or DADs. In line with the typical observation of prolonged APD and reduced repolarization reserve (Wang and Hill, 2010), in addition to the earlier described factors that possibly contribute to Na^+^ and Ca^2+^ overload in HF, it is likely that there is a reduced threshold for hypokalemia-induced afterdepolarizations in HF.

## Future targeted therapies for hypokalemia-induced triggered arrhythmias

No treatment that directly targets the underlying mechanism for hypokalemia-induced arrhythmias is currently available. The current treatment of hypokalemia in itself is potassium replacement (Cohn et al., 2000), and in patients with TDP, magnesium sulfate injection is used to prevent EADs and DADs through an unknown mechanism (Fazekas et al., 1993). TDP can also be treated with cardiac pacing or isoproterenol injection to shorten the APD (Banai and Tzivoni, 1993).

Ideally, new treatments against hypokalemia-induced arrhythmias should aim at (1) preventing EADs/DADs, (2) shortening the APD, and (3) directly targeting the underlying mechanism. On the basis of the model for hypokalemia-induced ventricular arrhythmias in Figure 2, we suggest CaMKII inhibition, NKA activation, and in particular NKAα2 activation, to be further investigated as future antiarrhythmic strategies. CaMKII inhibition prevents hypokalemia-induced EADs (Pezhouman et al., 2015), Ca^2+^ overload, and DADs in catecholaminergic polymorphic ventricular tachycardia (CPVT) (Di Pasquale et al., 2013) and in HF (Ai et al., 2005; Sag et al., 2009), in addition to hypokalemia-induced VT/VF (Pezhouman et al., 2015). CaMKII inhibition also shortens APD (Li et al., 2006; Bourgonje et al., 2012), although this effect might be species-dependent (Wagner et al., 2009). We are currently unaware of any drug that specifically activates the NKA. Theoretically, this could prove to be an effective antiarrhythmic strategy, as NKA inhibition causes Ca^2+^ overload, afterdepolarizations, and ventricular arrhythmias (Faggioni and Knollmann, 2015).

HF is also characterized by APD prolongation (Wang and Hill, 2010) and increased risk of afterdepolarizations (Pogwizd and Bers, 2004; Weiss et al., 2010), and we speculate that CaMKII inhibitors and NKA activators might be future antiarrhythmic options in HF even in the absence of hypokalemia.

## Author contributions

Both authors listed have made a substantial, direct and intellectual contribution to the work, and approved it for publication.

### Conflict of interest statement

The authors declare that the research was conducted in the absence of any commercial or financial relationships that could be construed as a potential conflict of interest.
